# Annual acknowledgement of manuscript reviewers

**DOI:** 10.1186/1471-2156-15-9

**Published:** 2014-03-06

**Authors:** Simon Harold

**Affiliations:** 1 BioMed Central, Floor 6, 236 Gray's Inn Road, London WC1X 8HB, UK

## Abstract

**Contributing reviewers:**

The editors of BMC Genetics would like to thank all of our reviewers who have contributed to the journal in Volume 14 (2013).

## 

Ian Adams

USA

Fernan Aguero

Argentina

Karen Aitken

Australia

Nurullah Akkoc

Turkey

Rafael G Albaladejo

Spain

Miguel Alcaide Torres

Canada

Santos Alonso

Spain

Stefan Ambs

USA

Marcel Amills

Spain

Leif Andersson

Sweden

Joaquin Arino

Spain

Melanie Bahlo

Australia

Gabriel Balmus

UK

Le Bao

USA

Marisa Bartolomei

USA

Andrea Bauer

Germany

Stephanie Baulac

France

Michael Baum

Tanzania

Dorothea Bedigian

USA

Tillmann Benfey

Canada

Gary Bennett

USA

Donagh Berry

Ireland

Soraya Bertioli

Brazil

Giorgio Binelli

Italy

Marco Bink

Netherlands

Elena Bitocchi

Italy

Zhu Bofeng

Croatia

James Bogart

Canada

Stefan Böhringer

Netherlands

Alicja Boron

Poland

Maria Bouga

Greece

Henk Bovenhuis

Netherlands

Neil Bowles

USA

Kevin Brix

Canada

Gudrun Brockmann

Germany

Heather Bruce

Canada

Anna Brüniche-Olsen

Australia

Greta Bunin

USA

John Burka

Canada

Diogo Cabral-De-Mello

Brazil

Lu Cai

USA

Mario Calus

Netherlands

Antonio Camargo

Spain

Paolo Carnier

Italy

Luigi Cattivelli

Italy

Salvatore Ceccarelli

Syria

Luigi Ceci

Italy

Giriraj Chandak

India

Peter Chandler

Australia

Hsueh-Wei Chang

Taiwan

Karen Chapman

UK

Michael Cheeseman

UK

Ji Yih Chen

Taiwan

Wei Chen

USA

Yy Chloe Cheung

Hong Kong

Ravindra Chibbar

Canada

Mingxing Chu

China

Mario Ciaffi

Italy

Marcelo Cioffi

Brazil

Guido Cipriani

Italy

Mete Civelek

USA

John Cole

USA

Catherine Collins

UK

Leonardo Congiu

Italy

Stuart Cook

Singapore

Antonio Costa De Oliveira

Brazil

Lindsay Crawford

Canada

Denny Crews

USA

John J Crowley

Ireland

Adam Croxford

Australia

Kehui Cui

China

Yuehua Cui

USA

Joaquim Culi

Spain

Robert Cushman

USA

Yang Da

USA

Kelsey Dancause

Canada

Richard Davis

USA

Roberta Davoli

Italy

Dirk-Jan De Koning

Sweden

Jan De Riek

Belgium

Jared Decker

USA

Nader Deeb

USA

François Delmotte

France

Dario Demarchi

Argentina

Olivier Demeure

France

Andrew Dewan

USA

Patrizio Dimitri

Italy

Ottmar Distl

Germany

Margaret Docker

Canada

Rajkumar Dorajoor

Singapore

David Douches

USA

Tom Druet

Belgium

Fang Du

China

Zhi-Qiang Du

USA

Monchai Duangjinda

Thailand

Ian Dunn

UK

Susana Dunner

Spain

Scott Edwards

USA

Julia Sarah El-Sayed Moustafa

UK

Sakina Elshibli

Finland

Francesco Emanuelli

Italy

Johannes Engelken

Spain

Michael Epstein

USA

Joan Estany

Spain

Justin Faris

USA

Laura Fejerman

USA

Jesús Fernández

Spain

Pedro Fernandez-Funez

USA

André Ferraz

Brazil

Sandra Fischer

Germany

Mark Fishbein

USA

Marie Fogarty

USA

Luca Fontanesi

Italy

Marina Fortes

Australia

Laurent Frantz

Netherlands

Merete Fredholm

Denmark

Matthias Frisch

Germany

Yong-Bi Fu

Canada

Takeshi Fukao

USA

Joseph Gall

USA

Indrajit Ganguly

India

Antonio Garcia

Spain

Daphne Gardner

Singapore

Dorian Garrick

USA

Mathieu Gautier

France

Hartwig H Geiger

Germany

Carine Genet

France

Fabrizio Ghiselli

Italy

Damjan Glavac

Slovenia

Luis Gomez-Raya

Spain

Eva Gonzales

USA

Boris Greber

Germany

Jieruo Gu

China

Francesca Gualandi

Italy

Carlos Guerrero-Bosagna

USA

Zhigang Guo

USA

Rajeev Gupta

India

Reinmar Hager

UK

Chris Haley

UK

Jiali Han

USA

Lide Han

USA

Olivier Hanotte

UK

Stephen Hartley

USA

Grit Haseneyer

Germany

Guohao He

USA

Tao He

USA

Xiaohong He

China

Jan Heggenes

Norway

James Hejtmancik

USA

Rasmus Heller

Portugal

Shirley Henderson

UK

Pilar Hernandez

Spain

Amaury Herpin

Germany

John Hickey

Australia

Ary Hoffmann

Australia

Josephine Hoh

USA

Mads Vilhelm Hollegaard

Denmark

Ross Houston

UK

Bjorn Hoyheim

Norway

Yi-Hsiang Hsu

USA

Yijuan Hu

USA

Yonghua Hu

China

Zhiqiu Hu

USA

Zhongli Hu

China

Ikhide Imumorin

USA

Wilfred Ip

Canada

Hiroyoshi Iwata

Japan

Nina Jablonski

USA

Mukesh Jain

India

Estelle Jaligot

France

Zhenyu Jia

USA

Feng Jianying

China

Anja Joachim

Austria

Elisabeth Jonas

Sweden

Hans-Georg Joost

Germany

Haja Kadarmideen

Denmark

Raj Kandpal

USA

Kyu-Suk Kang

Korea, South

Nevin Karakus

Turkey

Sanae Kasahara

Brazil

Ramesh Katam

USA

John William Keele

USA

Irene Keller

Switzerland

Matthew Kelly

Australia

Clement Kent

Canada

Susanne Kerje

Sweden

Chiea Chuen Khor

Singapore

Kitai Kim

USA

Etienne Klein

France

Yann Klimentidis

USA

Michael Hans Kohn

USA

Friedrich J Kopisch-Obuch

Germany

Alla Krasikova

Russian Federation

Michael Kristensen

Denmark

Per Kryger

Denmark

Eniko Kubinyi

Hungary

Gustavo Kuhn

Brazil

Mary Kuhner

USA

Anna Kukekova

USA

Anastasia Kulemzina

Russian Federation

Ravinder Kumar

India

Satish Kumar

New Zealand

Sunil Kumar

India

Valentina Kuznetsova

Russian Federation

Carles Lalueza-Fox

Spain

Susan Lamont

USA

Matthew Lanktree

Canada

Catherine Larzul

France

Hernan Laurentin

Venezuela

Massimiliano Lauria

Italy

Loic Le Cunff

France

Tosso Leeb

Switzerland

Sangram Keshari Lenka

USA

Johannes Lenstra

Netherlands

Stefano Leonardi

Italy

Ariadne Letra

USA

Chenxi Li

USA

Chun Li

China

Gengxin Li

USA

Huihui Li

China

Jiahan Li

USA

Ming Li

USA

Yi Li

Singapore

Zitong Li

Finland

Su-Chi Lim

Singapore

Daniela Lino Lourenco

USA

Bao Liu

China

Gaifen Liu

China

Jin Liu

USA

Sunny Liu

USA

Luciana Lourenco

Brazil

Qing Lu

USA

Tu Luan

Norway

Kerstin Ludwig

Germany

Philip Lupo

USA

Leslie Lyons

USA

Li Ma

USA

Dominique Madern

France

Shiro Maeda

Japan

Mauro Mandrioli

Italy

Ani Manichaikul

USA

Maurizio Margaglione

Italy

Daniela Marone

Italy

Paulino Martínez

Spain

Cesar Martins

Brazil

Anna Maria Mastrangelo

Italy

Angabin Matin

USA

Scot Matkovich

USA

Claudia Menzaghi

Italy

David Meyre

Canada

Thomas Miedaner

Germany

Satoshi Mikawa

Japan

Bill Milgram

Canada

Allison Miller

USA

Stephen Miller

Canada

Makiko Mimura

Japan

Bianca Moioli

Italy

Wagner Molina

Brazil

Samrat Mondol

India

Paul Moran

USA

Laurence Moreau

France

Garry Morgan

UK

Axel Muendlein

Austria

Louis Muglia

USA

James Nagler

USA

Indrajit Nanda

Germany

Holly Neibergs

USA

Stéphane Nicolas

France

Wen Hui Nie

China

Maen Obeidat

Canada

Konrad Ocalewicz

Poland

Gaetano Odierna

Italy

Devin Oglesbee

USA

Jun Ohashi

Japan

Junko Ohyashiki

Japan

Kenji Okumura

Japan

Klaus Oldach

Australia

Ettore Olmo

Italy

Hanne Gro Olsen

Norway

Rachel O'Neill

USA

Paul O'Reilly

UK

Pablo Orozco-Terwengel

UK

Jurg Ott

USA

Peggy Ozias-Akins

USA

Manish Pandey

India

Francisco Panzera

Uruguay

Heidi Parker

USA

Isela Parra-Rojas

Mexico

Marco Passamonti

Italy

Cameron Peace

USA

Nicola Pecchioni

Italy

David J Penman

UK

May Penrad-Mobayed

France

Alexandre Pereira

Brazil

Miguel Perez-Enciso

Spain

Andres Perez-Figueroa

Spain

Jessica Petersen

USA

Songsak Petmitr

Thailand

Davide Pettener

Italy

Marie-Hélène Pinard-Van Der Laan

France

Andrea Piotti

Italy

Eva Pisano

Italy

Monica Poelchau

USA

Francois Pompanon

France

Valerie Poncet

France

Ulrich K Posselt

Germany

Giorgio Prantera

Italy

Alex Pyron

USA

Xinshuai Qi

USA

Lijuan Qiu

China

Herman Raadsma

Australia

Petr Ráb

Czech Republic

Joe Rainger

UK

Harsh Raman

Australia

Domenico Rau

Italy

Bathini Mohan Reddy

India

Richard Redon

France

Jochen Reif

Germany

Juliette Riquet

France

Marylyn Ritchie

USA

Kelly Robbins

USA

Rebecca Roberts

New Zealand

Annie Robic

France

Raquel Rodríguez

Spain

Xavier Rognon

France

Gary Rohrer

USA

Megan Rolf

USA

Guilherme Rosa

USA

Garry Rosewarne

China

Alexandre Roulin

Switzerland

Suzanne Rowe

New Zealand

Oliver Ryder

USA

Richard Saffery

Australia

Goutam Sahana

Denmark

Yoshiro Saito

Japan

Magali Sancristobal

France

David Sargan

UK

Daniel J Sargent

Italy

Shinji Sasazaki

Japan

Eric Sayers

USA

Brian Sayre

USA

Massimo Scandura

Italy

John Schimenti

USA

Robert Schnabel

USA

James Schneider

USA

Ryohei Sekido

UK

Nick Serão

USA

Ramon Serrano

Spain

Qiuying Sha

USA

Abhay Sharma

India

Reeta Sharma

Portugal

Nadia Singh

USA

Montgomery Slatkin

USA

Petr Smykal

Czech Republic

Warren Snelling

USA

Catherine Sole

South Africa

Jiuzhou Song

USA

Tad Sonstegard

USA

Ivan Soto-Calderon

Colombia

Doug Speed

UK

Elizabeth Stacy

USA

Alessandra Stella

Italy

Don Stewart

Canada

Kathrin Friederike Stock

Germany

Ismo Stranden

Finland

Guosheng Su

Denmark

Prasanta Subudhi

USA

Liangdan Sun

China

Terje Svaasand

Norway

Akiko Takasuga

Japan

Aixin Tan

USA

Jifeng Tang

Netherlands

Marinus te Pas

Netherlands

Javier Terol

Spain

Milt Thomas

USA

Deborah Threadgill

USA

Gail Timmerman-Vaughan

New Zealand

Laurent Tiret

France

Chunfa Tong

China

Penny Tricker

Australia

David Tritchler

Canada

Tudorita Tumbar

USA

Stephen Turner

USA

Ryu Ueda

Japan

Yoshinobu Uemoto

Japan

Hirohide Uenishi

Japan

K Ulaganathan

India

Andreas Untergasser

Germany

Guilherme T Valente

Brazil

David Van Sanford

USA

George Vassiliou

UK

Roel Veerkamp

Netherlands

Thirumalaisamy Velavan

Germany

Juan Venegas

Chile

Patrick Venta

USA

Ricardo Ventura

Canada

Ignazio Verde

Italy

Ricardo Verdugo

USA

Yvonne Verkuil

Netherlands

Fabio Veronesi

Italy

Hans-Henning Voss

USA

Ronald Walter

USA

Chenguang Wang

USA

Chunkao Wang

USA

Kai Wang

USA

Ming Li Wang

USA

Xuefeng Wang

USA

Yaqun Wang

USA

Zuoheng Wang

USA

Toshio Watanabe

Japan

David Weatherall

UK

Daniel Wegmann

Switzerland

Peng Wei

USA

Kent Weigel

Italy

Steffen Weigend

Germany

Robert Weiss

USA

Stephan Wessels

France

Stephen White

USA

John Wiens

USA

Klaus Wimmers

Germany

Charles Wondji

UK

Naomi Wray

Australia

Xiaowei Wu

USA

Ying Wu

USA

Bo Xi

China

Yongzhong Xing

China

Wang Xiuli

China

Haiming Xu

China

Jianping Xu

Canada

Li Xu

USA

Shuhua Xu

China

Eiji Yamamoto

Japan

Hsin-Chou Yang

Taiwan

Jie Yang

USA

Chaoqun Yao

USA

Sibin Yu

China

Gen Hua Yue

Singapore

Junming Yue

USA

Sabrina Yum

USA

Guo Yunqian

China

Aimin Zhang

China

Haitao Zhang

USA

Jing Zhang

USA

Qin Zhang

China

Xi Zhang

Singapore

Xiquan Zhang

China

Yue-Ran Zhao

China

Bing Song Zheng

China

Min Zhong

USA

Chengsong Zhu

USA

Hongxiao Zhu

USA

Xiaofeng Zhu

USA

Cheng Zou

China

